# Physician perspectives on the management of viral hepatitis and hepatocellular carcinoma in Myanmar

**DOI:** 10.1371/journal.pone.0181603

**Published:** 2017-08-10

**Authors:** Yoona A. Kim, Sam Trinh, Si Thura, Khin Pyone Kyi, Thomas Lee, Stan Sze, Adam Richards, Andrew Aronsohn, Grace L. H. Wong, Yasuhito Tanaka, Geoffrey Dusheiko, Mindie H. Nguyen

**Affiliations:** 1 Division of Gastroenterology and Hepatology, Stanford University, Stanford, California, United States of America; 2 Community Partners International, Berkeley, California, United States of America; 3 Myanmar Liver Foundation, Yangon, Myanmar; 4 Department of Emergency Medicine, University of California Los Angeles, Los Angeles, California, United States of America; 5 B.K. Kee Foundation, Yangon, Myanmar; 6 Division of General Internal Medicine and Health Services Research, University of California Los Angeles, Los Angeles, California, United States of America; 7 Division of Gastroenterology and Hepatology, University of Chicago Medical Center, Chicago, Illinois, United States of America; 8 Institute of Digestive Disease and Department of Medicine & Therapeutics, The Chinese University of Hong Kong, Hong Kong, Peoples Republic of China; 9 Nagoya City University Graduate School of Medical Sciences, Nagoya, Japan; 10 Kings College Hospital and University College School of Medicine, London, United Kingdom; Kaohsiung Medical University Chung Ho Memorial Hospital, TAIWAN

## Abstract

**Background:**

In Myanmar, over five million people are infected with hepatitis B virus (HBV) and hepatitis C virus (HCV). Hepatitis has been a recent focus with the development of a National Strategic Plan on Hepatitis and plans to subsidize HCV treatment.

**Methods:**

During a two-day national liver disease symposium covering HCV, HBV, hepatocellular (HCC), and end-stage liver disease (ESLD), physician surveys were administered using the automated response system (ARS) to assess physician knowledge, perceptions of barriers to screening and treatment, and proposed solutions. Multivariate logistic regression was used to estimate odds ratio (OR) relating demography and practice factors with higher provider knowledge and improvement.

**Results:**

One hundred two physicians attending from various specialty areas (31.0% specializing in gastroenterology/hepatology and/or infectious disease) were of mixed gender (46.8% male), were younger than or equal to 40 years old (51.1% 20 to 40 years), had less experience (61.6% with ≤10 years of medical practice), were from the metropolitan area of Yangon (72.1%), and saw <10 liver disease patients per week (74.3%). The majority of physicians were not comfortable with treating or managing patients with liver disease. The post-test scores demonstrated an improvement in liver disease knowledge (9.0% ± 27.0) compared to the baseline pre-test scores; no variables were associated with significant improvement in hepatitis knowledge. Physicians identified the cost of diagnostic blood tests and treatment as the most significant barrier to treatment. Top solutions proposed were universal screening policies (46%), removal of financial barriers for treatment (29%), patient education (14%) and provider education (11%).

**Conclusions:**

Physician knowledge improved after this symposium, and many other needs were revealed by the physician input on barriers to care and their solutions. These survey results are important in guiding the next steps to improve liver disease management and future medical education efforts in Myanmar.

## Introduction

A recent nationwide prevalence study revealed that nearly 5 million persons in Myanmar are affected by viral hepatitis B (HBV) and C (HCV) [1]. The goal of the study was to provide evidence-based prevalence data to the National Hepatitis Control Program (NHCP), which in turn would allow the NHCP committee to develop recommendations for policymakers on reducing the hepatitis disease burden. The cross-sectional study was conducted in the general Myanmar population (n = 5547) which included 18 townships and peoples aged 15 to 80 years from May to November 2015. The average prevalence rate for HBV was found to be 6.5%, while the average prevalence for HCV was 2.65% [1].

Viral hepatitis has long been recognized in Myanmar, with the earliest research conducted in 1958 [2]. In 2000, efforts to screen the blood donor population were initiated, as well as the promotion and expansion of HBV immunization, including vaccination at birth, was initiated in 2003 [3,4].

Viral hepatitis management has also been a recent focus in Myanmar. In June 2014, the Myanmar Gastroenterology and Liver Society revised the 2009 guidelines for the treatment of chronic HCV to provide practical management of HCV infection. These revised guidelines were simplified, with the intent to allow treatment to be prescribed by most doctors for all HCV patients. In September 2015, discussions began on the development of a National Strategic Plan on Hepatitis. From these discussions and in collaboration with the Ministry of Health and Sports (MOHS), the Myanmar Health Sector Coordinating Committee (MHSCC), non-governmental organizations (NGOs) and the United Nations (UN), efforts will begin in 2017 to treat 2,000 HCV-infected patients with daclastavir and sofosbuvir in Yangon and Mandalay [5,6].

In order to prepare for the successful initiation of treatment with the new direct acting antiviral agents (DAAs) (daclastavir and sofosbuvir) for HCV as well as general advancement of liver disease management in Myanmar, several goals were proposed, such as understanding the current level of physician knowledge on the management of HCV in Myanmar, ascertaining physician comfort level in the management of liver disease, and delineating the barriers to screening, diagnosis and treatment and subsequent proposed solutions.

## Methods

### Study design

The B. K. Kee Foundation, Stanford University, and the MOHS conducted a two-day viral hepatitis symposium from October 31 to November 1, 2016 in Yangon, Myanmar, where international experts in liver disease led interactive sessions on HBV and HCV, special populations such as the pregnant and co-infected, as well as end-stage liver disease (ESLD) and hepatocellular carcinoma (HCC).

During the symposium, local hepatologists moderated case study discussions after each module and administered anonymous surveys in the Burmese language. Surveys were administered at regular intervals throughout the sessions to evaluate physicians’ knowledge of liver disease, current screening and treatment rates, physician comfort level in the management of liver disease, perceived barriers to screening, diagnosis, and treatment as well as suggested solutions to these problems.

Paper surveys were administered for the demographic and practice questions, while an audience response system (ARS) was used to display, record, and tabulate responses. Physicians’ demographics were matched to the ARS by recording the ARS handheld device number on the paper surveys. All written materials, including paper survey, slides of survey questions, lecture slides and course syllabus, were presented in both the English and Burmese languages. Burmese translation of the lectures was available via headset. The suggested solution responses were based on a ranking scale of 1 to 5, with 5 representing the most effective and 1 the least effective.

### Statistical analysis

Summary statistics were performed on all surveys using STATA (StataCorp LP, College Station, Texas, USA). A composite score of hepatitis knowledge was calculated for each individual who answered at least one question as the ratio of the number of correct responses to the total number of questions. We considered a ten percentage point increase in hepatitis knowledge to be clinically meaningful, and we used multivariate logistic regression models to estimate odds ratio (OR) of dichotomous outcome of 10% improvement in hepatitis knowledge score, controlling for relating factors such as physician gender, age (≤30 or >30 years), years of practice (<10 or ≥10 years), percent of time devoted to patient care (<50% or ≥50%), and university affiliation. This study received an Institutional Review Board exemption from the Panel on Human Subjects at Stanford University, Stanford, California, USA and was approved by the Ethical Review Committee of the Department of Medical Research, Myanmar Ministry of Health and Sports, Republic of Union of Myanmar. The participants were given an instruction sheet explaining the research study, risks and benefits, subject’s right to withdraw consent and statement that they will receive no payment for their anonymized participation. The form stated that agreement to participate in this research was indicated by completing the survey.

## Results

### Demographics

Out of the total of 102 attendees, 76 participated in the surveys and the average response rate was 48.2% ± 13.1% for the 203 survey questions. The participating physicians were from various specialty areas (31.0% specializing in gastroenterology/hepatology and/or infectious disease), mixed gender (46.8% male), half were younger than or equal to 40 years old (51.1% 20–40 years, 32.0% 41–60 years, 17.0% >60 years), and with most having less than 10 years’ experience in medical practice (61.6%). The majority of physicians (72.1%) were from the metropolitan area of Yangon and only 4.7% were from rural areas of Myanmar. Most of the physicians (61.4%) spent ≤50% of their time on patient care, while 43.6% spent ≥25% on teaching. The majority of the physicians (74.3%) saw <10 liver disease patients per week (Table 1).

**Table 1:** Participant demographics.

Characteristic	n (%)	
**Gender (n = 47)**		
Male	22 (46.8%)	
Female	25 (53.2%)	
**Age range (n = 47)**		
20–30 years	14 (29.8%)	
31–40 years	10 (21.3%)	
41–50 years	13 (27.7%)	
51–60 years	2 (4.3%)	
61–65 years	5 (10.6%)	
66–70 years	3 (6.4%)	
≥ 71 years	0 (0%)	
**Years of practice (n = 39)**		
0–5 years	17 (43.6%)	
5–10 years	7 (18.0%)	
11–20 years	8 (20.5%)	
>20 years	7 (18.0%)	
**Percent of time devoted to patient care (n = 44)**		
None	2 (4.6%)	
<25%	10 (22.7%)	
25–50%	15 (34.1%)	
51–75%	14 (31.8%)	
75–100%	3 (6.8%)	
**Percent of time devoted to research (n = 46)**		
None	12 (26.1%)	
<25%	17 (37.0%)	
25–50%	8 (17.4%)	
50–75%	3 (6.5%)	
75–100%	6 (13.0%)	
**Percent of time devoted to teaching (n = 46)**		
None	7 (15.2%)	
<25%	19 (41.3%)	
25–50%	13 (28.3%)	
50–75%	5 (10.9%)	
75–100%	2 (4.4%)	
**Specialty (n = 45)**		
Family Medicine	1 (2.2%)	
Internal Medicine	10 (22.2%)	
Gastroenterology	0 (0%)	
Hepatology	5 (11.1%)	
Infectious Disease	5 (11.1%)	
OB/GYN	0 (0%)	
Pediatrics	1 (2.2%)	
Other	17 (37.8%)	
**More than one specialty:**	6 (13.3%)	
Internal + Hepatology	1 (2.2%)	
Internal + Other	2 (4.4%)	
Gastroenterology + Hepatology	1 (2.2%)	
Hepatology + Infectious Disease	1 (2.2%)	
Gastroenterology + Hepatology + Other	1 (2.2%)	
**Primary medicine practice (n = 47)**		
Referral government hospital	6 (13.3%)	
General government hospital	30 (66.7%)	
Referral private hospital	0 (0%)	
General private hospital	1 (2.2%)	
Referral public clinic	0 (0%)	
General primary care public clinic	0 (0%)	
Referral private clinic	0 (0%)	
General practice private clinic	6 (13.3%)	
**More than one practice:**	2 (4.4%)	
[Referral + General government hospital + General private clinic]	1 (2.2%)	
[Referral + General government hospital]	1 (2.2%)	
**University affiliation (n = 46)**		
Yes–university on campus/trainees involved		23 (50.0%)
Yes–university unattached/trainees not involved		4 (8.7%)
Not affiliated		19 (41.3%)
**Size of hospital facility (n = 46)**		
≥500 bed hospital		4 (8.7%)
300–500 bed hospital		1 (2.2%)
100–300 bed hospital		5 (10.9%)
≤100 bed hospital		4 (8.7%)
Does not work in the hospital		32 (69.6%)
**Size of physician clinic facility (n = 44)**		
Single physician clinic		10 (22.7%)
2–5 physician clinic		6 (13.6%)
6–10 physician clinic		4 (9.1%)
≥11 physician clinic		10 (22.7%)
Does not work in the clinic		14 (31.8%)
**Area of Myanmar (n = 43)**		
Yangon		31 (72.1%)
Other large cities (>100,000 population)		7 (16.3%)
Small cities (<100,000 population)		3 (7.0%)
Rural		2 (4.7%)
**Patients with liver disease seen per week (n = 35)**		
<10		26 (74.3%)
11–30		2 (5.7%)
31–50		1 (2.9%)
51–75		0 (0%)
76–100		1 (2.9%)
101–150		2 (5.7%)
>150		3 (8.6%)

### Screening and treatment rates

Reported HBV and HCV screening rates were low, with 53.7% and 69.0% of physicians observing that ≤25% of patients in need of screening receive screening for HBV and HCV, respectively. The screening rates for HCC were similarly low: 71.2% of physicians noted that ≤25% of patients in need of screening receive it. Reported treatment rates were also low, as 68% of physicians treated ≤10 patients for HCV in the last 3 months, 79% indicated that ≤10% of high priority patients received treatment, and 76% of physicians treated <25% of these patients with the new DAAs. Similarly, 66% of providers treated ≤10 patients for chronic HBV in the past 3 months, and 49% of physicians indicated that only ≤10% of patients qualifying for treatment received it. Reported newborn vaccination rates for HBV were higher; 42% of providers noted that >51% of newborns receive HBV vaccinations. HCC treatment rates were low, with 63% of physicians noting that only ≤10 patients with HCC receive treatment.

### Comfort level with managing liver disease

Most physicians (73.4%) were aware of the new HCV DAA treatments, but only 45.6% felt comfortable or very comfortable with initiating and monitoring HCV treatment. Surprisingly, even fewer physicians (26.9%) were comfortable or very comfortable with merely monitoring patients with HCV. These same trends held true for physicians’ comfort in monitoring and treating patients with HBV, HCC, cirrhosis, and ESLD (percent comfortable treating HBV 35.3%, HCC 22.5%, cirrhosis 37.1%, and ESLD 20%). Interestingly, this cross-sectional group of physicians were most comfortable monitoring and treating co-infected patients (52.3%) (Table 2).

**Table 2:** Physician’s level of comfort managing and monitoring patients with liver disease.

**Please rate your comfort level in initiating and monitoring hepatitis C treatment (n = 46)**	
Not comfortable	5 (10.9%)
Somewhat comfortable	7 (15.2%)
Neutral	13 (28.3%)
Comfortable	18 (39.1%)
Very comfortable	3 (6.5%)
**Please rate your comfort level in monitoring hepatitis C patients not on treatment (n = 41)**	
Not comfortable	13 (31.7%)
Somewhat comfortable	9 (22.0%)
Neutral	8 (19.5%)
Comfortable	9 (22.0%)
Very comfortable	2 (4.9%)
**Please rate your comfort level in monitoring hepatitis B patients not on treatment (n = 26)**	
Not comfortable	4 (15.4%)
Somewhat comfortable	4 (15.4%)
Neutral	9 (34.6%)
Comfortable	7 (26.9%)
Very comfortable	3 (7.7%)
**Please rate your comfort level in initiating and monitoring hepatitis B treatment (n = 34)**	
Not comfortable	7 (20.6%)
Somewhat comfortable	5 (14.7%)
Neutral	10 (29.4%)
Comfortable	7 (20.6%)
Very comfortable	5 (14.7%)
**Please rate your comfort level in providing medical therapy for HCC treatment (n = 40)**	
Not comfortable	14 (35.0%)
Somewhat comfortable	9 (22.5%)
Neutral	12 (30.0%)
Comfortable	4 (10.0%)
Very comfortable	1 (2.5%)
**Please rate your comfort level in monitoring HIV/HCV co-infected patients (n = 44)**	
Not comfortable	8 (18.2%)
Somewhat comfortable	4 (9.1%)
Neutral	5 (11.4%)
Comfortable	23 (52.3%)
Very comfortable	4 (9.1%)
**Please rate your comfort level in monitoring patients with cirrhosis (n = 35)**	
Not comfortable	2 (5.7%)
Somewhat comfortable	5 (14.3%)
Neutral	13 (37.1%)
Comfortable	13 (37.1%)
Very comfortable	2 (5.7%)
**Please rate your comfort level in managing patients with ESLD (n = 40)**	
Not comfortable	16 (40.0%)
Somewhat comfortable	4 (10.0%)
Neutral	1 (27.5%)
Comfortable	8 (20.0%)
Very Comfortable	1 (2.5%)
**Please rate your awareness of available HCV drugs (n = 47)**	
Not aware	4 (8.5%)
Somewhat aware	9 (19.2%)
Aware	24 (51.1%)
Very aware	10 (21.3%)

### Physician scores on knowledge-based questions

The mean pre-survey test score was 35.4% ± 22.1 compared to a post-survey score of 43.0% ± 18.5, showing an improvement of 9.0% ± 27.0. Approximately 31.6% of participants improved their score by at least 10 percentage points. In a multivariate model, no factors were associated with a ≥10% improvement in the pre-test vs. post-test scores, but gender was borderline significant (F vs. M OR: 5.52, 95% CI: 0.99–30.9). In general, physicians struggled with the HBV, HCV, and cirrhosis case study questions, scoring less than a mean score of 40% per section (31.8% ± 19.1, 39.0% ± 26, 36.5% ± 20.7, respectively). However, the physicians had highest scores (50.0% ± 17.6) in the special population section, suggesting that they have better knowledge of mother-to-child transmission of HBV (Table 3).

**Table 3:** Hepatitis knowledge score (% correct answers on knowledge-based questions).

Parameter	Mean % Correct ± Standard Deviation
**Pre-survey (mean ± SD)**	
Overall score (n = 76)	35.4 ± 22.1
**Post-survey (mean ± SD)**	
Overall score (n = 55)	43.0 ± 18.5
**Change in score (n = 44)**	**9.0 ± 27.0**
**Case studies (mean ± SD)**	
HBV (n = 66)	31.8 ± 19.1
HCV (n = 47)	39.0 ± 26.4
Special populations (n = 60)	50.0 ± 17.6
Cirrhosis (n = 66)	36.5 ± 20.7
**Total score across sessions (n = 23)**	**43.0 ± 14.1**

### Perceived barriers to liver disease management

**Fig 1:**
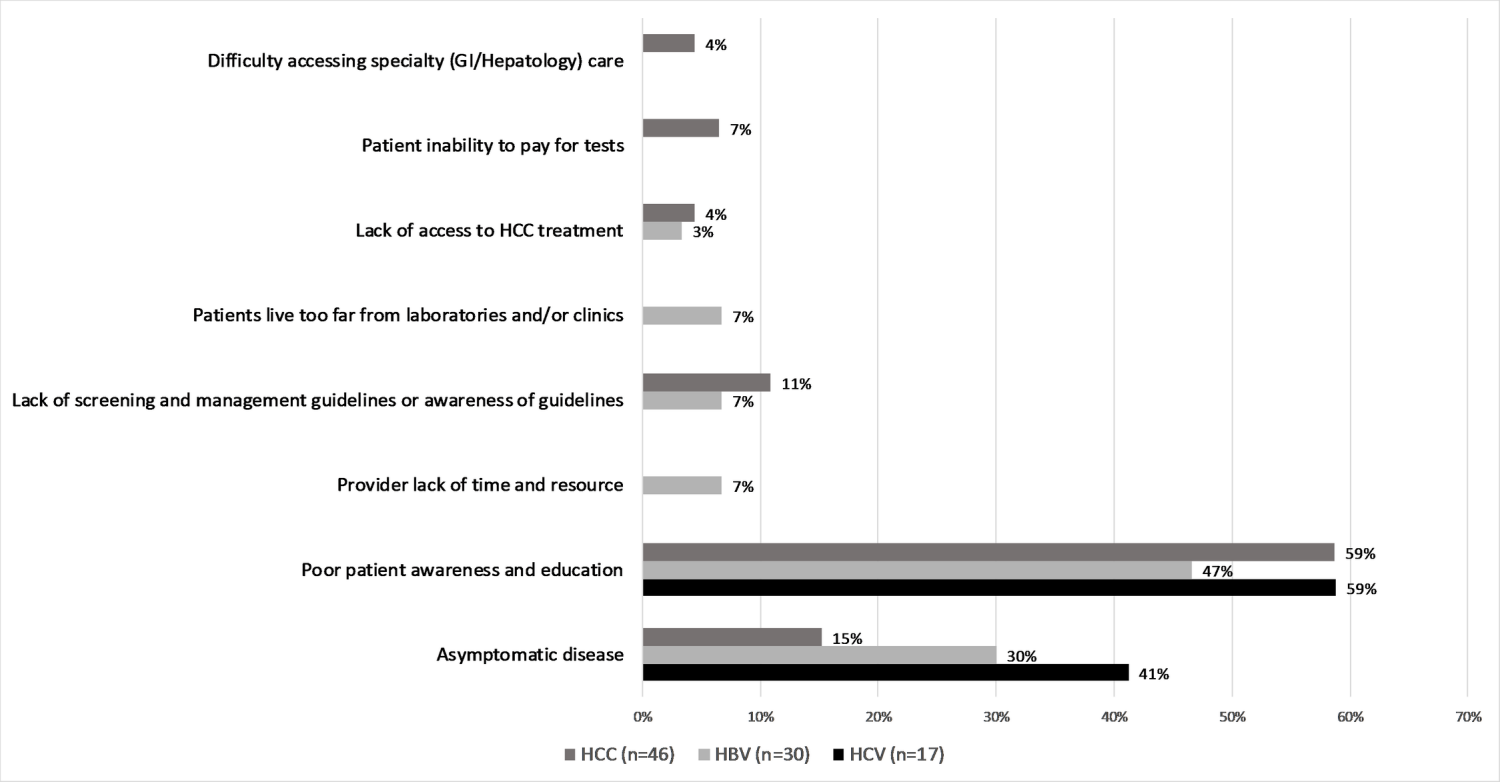
Perceived barriers to liver disease management.

The top barriers to screening were reported as poor patient awareness and education and asymptomatic disease for all liver disease categories (HCC, HBV, and HCV) (Fig 1).

**Fig 2:**
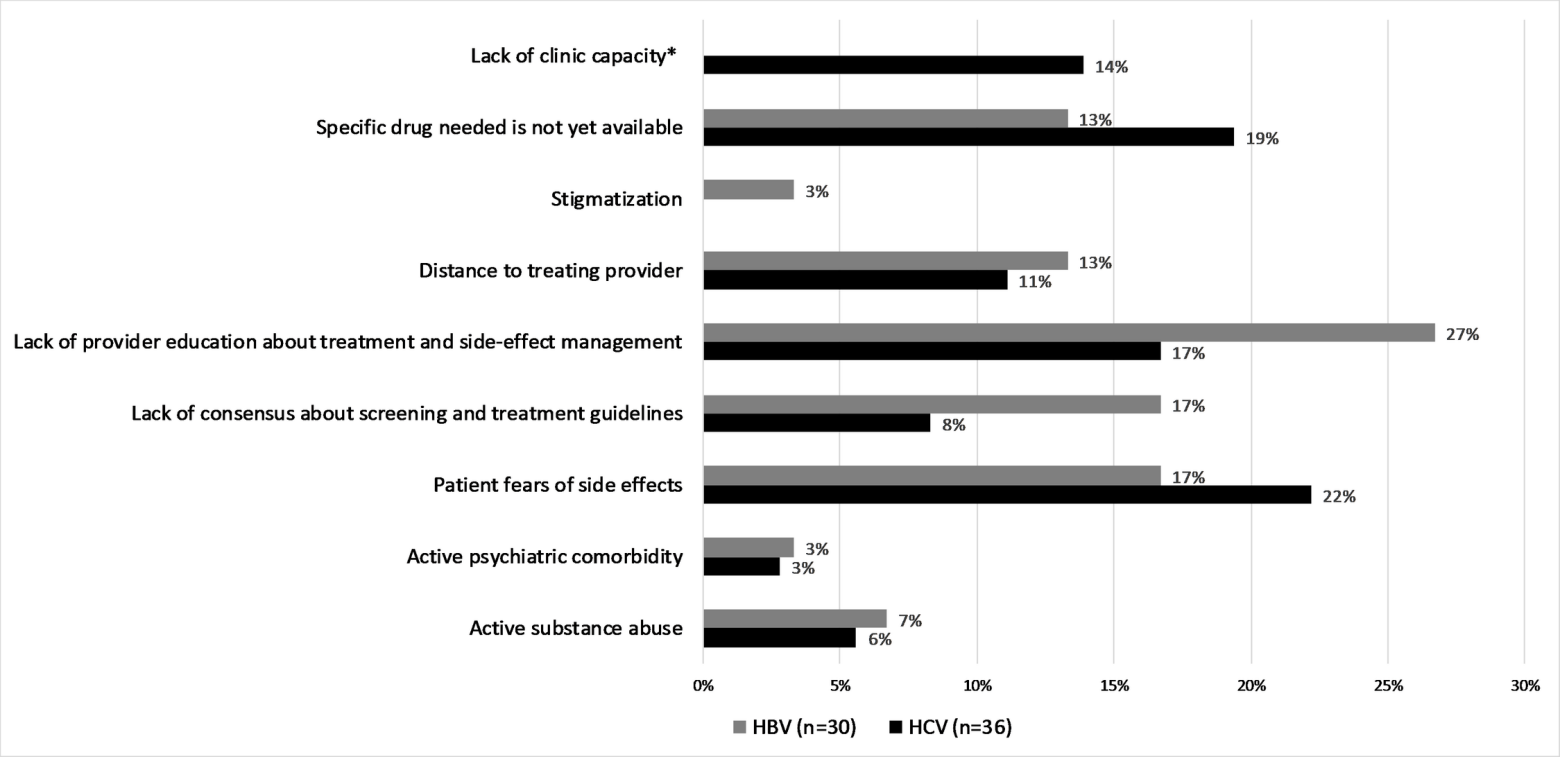
Barriers to HBV and HCV disease management.

The principal barrier to treatment was clearly the cost of medications and blood tests, with 64% and 38% of physicians noting costs of medications as the top barrier for HCV and HBV, respectively, while 26% and 43% of physicians noted costs of medical tests and visits as the top barrier for HCV and HBV, respectively. The other barriers for HBV besides costs of treatment included lack of provider education about treatment and side-effect management (27%), lack of clinical guidelines (17%), and patient fears of medication side-effects (17%). For HCV, the top three barriers other than costs included patient fear of medication side-effects (22%), lack of medicine availability (19%), and lack of provider education about treatment (17) (Fig 2).

#### Physician-suggested proposed solutions

When asked for the top solutions to ensure better liver disease management, the major physician responses included implementation of universal screening policies (46%), removal of treatment financial barriers (29%), patient education about liver disease (14%), and enhancement of provider education (11%).

**Fig 3:**
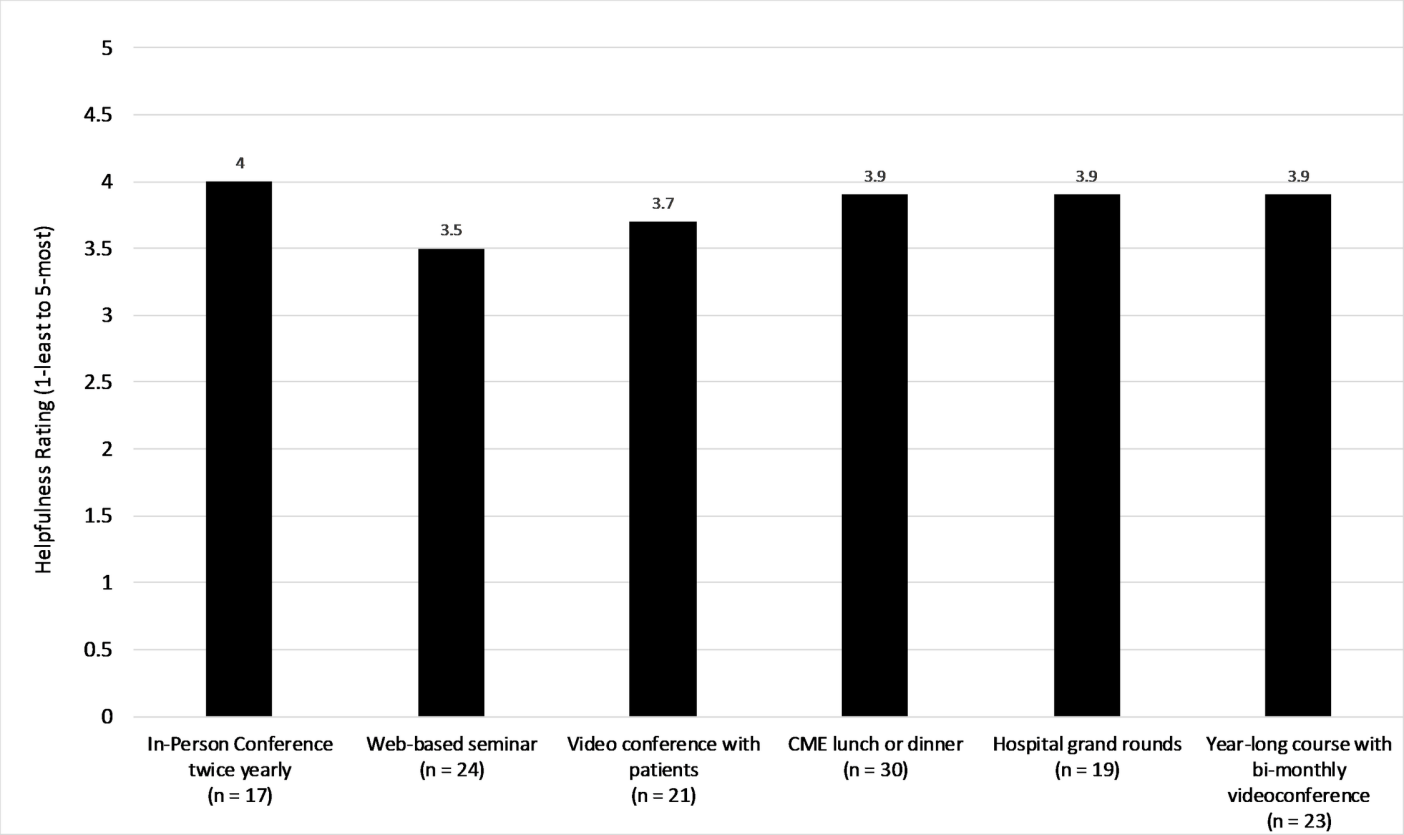
Physician education choices for liver disease management.

To improve physicians’ skills in managing liver disease, the physicians ranked an in-person conference two times a year as the most important (4), followed by offering continuing medical education (CME) lunches and/or dinners (3.9), having hospital grand rounds (3.9), and offering a year-long course with bimonthly video conferencing (3.9) (scale 1–5, with 5 the most effective and 1 the least effective) (Fig 3).

**Fig 4:**
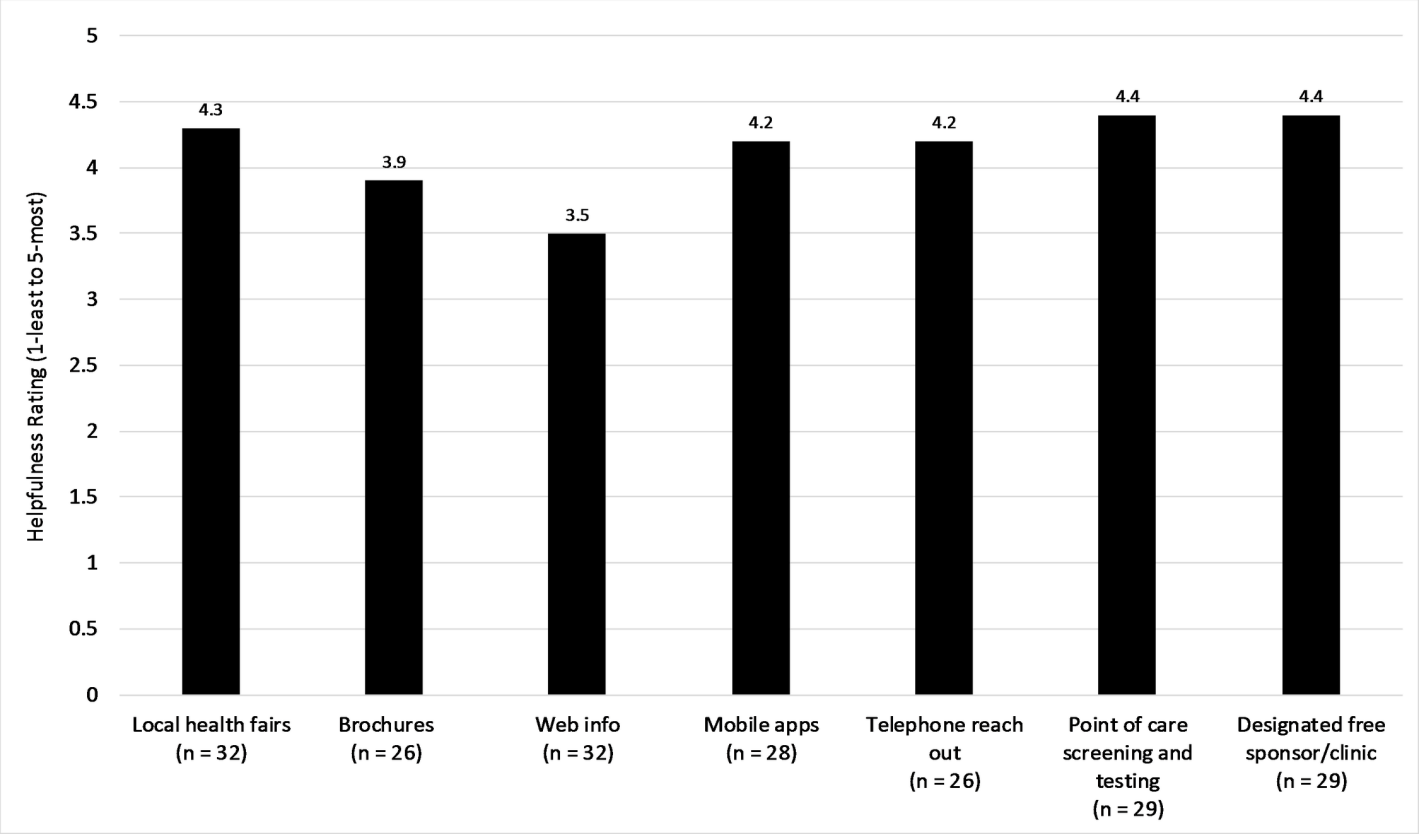
Preferred physician interventions to improve education and screening for liver disease management.

When physicians were asked to rank a list of interventions/tools to help physicians provide education and screening to the general population, the top responses provided were point-of-care testing (4.4), designated free clinics (4.4), local health fairs (4.3), mobile education applications (4.2), and telephone outreach programs (4.2) (Fig 4).

## Discussion

The purpose of this study was to obtain an understanding of physician knowledge, comfort level, perceived barriers, and proposed solutions for the management of viral hepatitis-related liver disease, and to inform policymakers and researchers in the National Program on Hepatitis Control in Myanmar. Each country has their own specific needs, and barriers to care differ accordingly, emphasizing the importance of obtaining feedback from the frontline local physicians [7]. The success of the symposium and survey was evident by the support of the MOHS, the attendance of policymakers and researchers from the Liver Disease Foundation, high physician attendance, and improvement in knowledge-based questions. Such support from the key policymakers is especially important as Myanmar has been challenged with fragmentation and unsustainable efforts due to funding mechanisms which have largely bypassed government sources, according to a report on the Myanmar health system [8]. In addition to the support of the symposium, knowledge for the policymakers and researchers was shared to help advance the management of hepatitis-related liver disease in Myanmar. There are several recommendations stemming from the survey results and symposium.

Concerted efforts should be made to include rural physicians in future educational events. The symposium was intended to target treating physicians across the country, but many of the participants (61.4%) spent ≤50% of their time on patient care and the large majority (72.1%) were from Yangon, a large metropolitan area. In addition, the majority of physicians (74.3%) saw only <10 liver disease patients per week. The same format of symposium was given in Mongolia in 2015 with a very different audience from all the major provinces and 38.8% from the rural areas [9]. Furthermore, in contrast to the Burmese attendees, the majority of whom managed <10 patients per week and were not specialists, 78.1% of the Mongolian physicians managed ≥10 patients per week with liver disease and 81.1% were sub-specialists [9]. In many ways, the audience is reflective of the disparities in Myanmar, with attendees mostly from larger institutions in Yangon rather than treating physicians from rural areas attending the symposium. In this country of 51.4 million people, 70% live in rural areas and poverty is twice as high in these areas [10]. Access to healthcare among the poor is especially challenging, since the private sector is the dominant source of health financing accounting for more than 80% of total health expenditures, and there is no government subsidy for the poor [8,11]. Hepatitis drugs are not currently subsidized by the government except for the new effort to treat 2,000 HCV-infected patients with daclastavir and sofosbuvir in Yangon and Mandalay [5,6].

Given these disparities, future medical education symposiums should be held locally or include options the rural healthcare workforce and organizations to join remotely. The number one preferred method of education was having an in-person conference two times a year, followed by offering CME lunches and/or dinners, having hospital grand rounds, and offering a year-long course with bimonthly video conferencing. One training model that may be used is based on the “Rural Physicians Action Plan in Alberta” [12]. This plan is based on the better-known concept of “train the trainer,” a method that has been proven to be an excellent method way to provide education to wide and diverse populations. “Train the trainer” is based on using “one of their own”—in this case rural community physicians to advocate on behalf of local doctors and who would organize training programs to meet the needs of rural communities. In fact, studies which have used this concept have had great success in reaching and training rural physicians. Follow-up has shown significant knowledge retention in most areas of training, but does suggest that rural physicians need a mechanism to reach out to experts when dealing with more difficult patients [13,14,15]. This type of advocacy for education may also lead to improved retention of healthcare providers in the rural areas of Myanmar, which is disadvantaged by a shortage of healthcare workers [8]. Another option is to use remote educational and mentorship program, such as Project ECHO. This project was presented at the symposium and provides access to specialty care and consultation for patients and physicians in rural areas. One study found that the quality of hepatitis C care provided by ECHO-trained primary care providers was equivalent to care provided by university-based specialists.[16] Furthermore, any educational efforts should be extended to non-physician workforce, such as health assistants and midwives, who provide the majority of healthcare services in the rural areas. [11] Involvement from and education of non-governmental organizations, ethnic health organizations, and private-for-profit providers are also key. The Myanmar National Health Plan, which aims to pave the way to universal health coverage in Myanmar, specifically calls out the need to engage organizations outside of the public sector to strengthen the country’s health system. [17]

Another need identified by the physicians was HBV treatment guidelines. Scores were lowest on the HBV case studies (31.8% correct) vs. HCV and ESLD, and 27% of physicians ranked “lack of provider knowledge about treatment and side effect management” as a top perceived barrier to treatment. The HCV guidelines were recently updated in 2016, and increased focus has been given to HCV, given the new DAA therapies. However, HBV is noted by 50% of physicians as the number one cause of HCC in Myanmar (compared to 33.3% citing HCV and 16.7% citing alcoholic cirrhosis as the main cause). Consequently, development of HBV guidelines should be considered and further training on HBV management should be provided.

The most important solution to address gaps in current management proposed by the physicians was universal screening. One example of a universal policy in Myanmar with important lessons learned was the national HBV vaccination program. HBV vaccine was first introduced in 2003, but the program ended in 2008–2009 due to the primary reliance on external funding [18]. The government was able to co-finance the cost of Pentavalent vaccines (DPT + Haemophilus influenza + HBV) in 2012, and also launched a year of Intensification of routine immunization to reduce gaps in coverage for more equitable access [18]. Regarding universal screening for hepatitis, costs were noted as a key barrier by the physicians. Rapid point-of-care testing kits (US $0.50-$2) and liver function tests (approximately US$20 each) used to diagnose hepatitis are significant and often insurmountable expenses for most of the population [19]. Based on the HBV vaccine experience, government co-financing and commitment would be vital to implement a sustainable and effective policy for hepatitis screening. Barriers to implementation can arise when clear government priorities are not set or when wholly dependent on foreign aid, which can stop abruptly for various reasons [8]. Screening can identify high-risk patients and would also produce the demographic information necessary to prioritize patients for treatment. Furthermore, when coupled with surveillance, targeted screening efforts could be used to identify sources and modes of virus transmission and design prevention interventions to reduce incidence further infection.

Another aspect of encouraging screening is to increase patient awareness and knowledge of hepatic disease. The physicians ranked point-of-care screening and testing (4.4 out of 5.0), designated free sponsors or clinics (4.4) and local health fairs (4.3) as the top three solutions to improve screening rates. As with physicians, local personal efforts seem to be preferred over web-based solutions. As only 2 out of 100 people have access to the internet in Myanmar [20], the needs and access challenges of rural communities should be at the forefront when planning screening initiatives.

Limitations of this study include the lack of diversity among the physician group. The symposium was intended to target treating physicians across the country, but the majority came from urban areas (from Yangon specifically) with limited rural representation. In addition, the majority treated very few liver disease patients a month, many had less than 10 years of medical experience, and many spent less than 50% of their time in clinical settings. Also, only physicians were present, despite the key role of other provider types, such as community healthcare workers. The response rate at 48.2% ± 13.1% could have been higher, but many questions (203 questions total, most with multiple choices) and topics were covered in the surveys. Despite these limitations, the surveys did allow for accurate physician perspective on caring for liver disease patients.

## Conclusions

This is the first survey capturing physician perceptions of liver disease management in Myanmar. The political, economic, and social context of the country as well as perceptions of the front-line physicians should be considered in order to develop effective proposals and guidelines to improve liver disease management in Myanmar. The responses indicated the need to expand medical educational programs to include provider teams from rural communities with a high burden of liver disease. There is increased momentum around this topic with MOHS support, and this momentum should stimulate government linkage of grass roots efforts to ensure that education and treatment of liver diseases becomes more readily available.
